# Biochemical and physiological insights into TRH receptor-mediated signaling

**DOI:** 10.3389/fcell.2022.981452

**Published:** 2022-09-06

**Authors:** Radka Trubacova, Zdenka Drastichova, Jiri Novotny

**Affiliations:** Department of Physiology, Faculty of Science, Charles University, Prague, Czechia

**Keywords:** thyrotropin-releasing hormone, TRH receptors, G protein, β-arrestin, signaling

## Abstract

Thyrotropin-releasing hormone (TRH) is an important endocrine agent that regulates the function of cells in the anterior pituitary and the central and peripheral nervous systems. By controlling the synthesis and release of thyroid hormones, TRH affects many physiological functions, including energy homeostasis. This hormone exerts its effects through G protein-coupled TRH receptors, which signal primarily through G_q/11_ but may also utilize other G protein classes under certain conditions. Because of the potential therapeutic benefit, considerable attention has been devoted to the synthesis of new TRH analogs that may have some advantageous properties compared with TRH. In this context, it may be interesting to consider the phenomenon of biased agonism and signaling at the TRH receptor. This possibility is supported by some recent findings. Although knowledge about the mechanisms of TRH receptor-mediated signaling has increased steadily over the past decades, there are still many unanswered questions, particularly about the molecular details of post-receptor signaling. In this review, we summarize what has been learned to date about TRH receptor-mediated signaling, including some previously undiscussed information, and point to future directions in TRH research that may offer new insights into the molecular mechanisms of TRH receptor-triggered actions and possible ways to modulate TRH receptor-mediated signaling.

## Introduction

Thyrotropin-releasing hormone (TRH), also termed thyroliberin, is a tripeptide (pGlu-His-Pro-NH_2_) hormone primarily involved in regulating pituitary function. Its main role is to maintain thyroid hormone homeostasis through regulation of thyroid-stimulating hormone secretion (Hoermann et al., 2015). However, TRH may cause different non-thyroidal effects as well (Fröhlich and Wahl, 2019). It has been observed that TRH is involved in the regulation of thermogenesis, feeding behavior and water intake (Choi et al., 2002), and it can play a role in the pathophysiology of major depression disorder (Tsuru et al., 2013). TRH as well as TRH receptors (TRH-R) are conserved from man to bony fish demonstrating the importance of this peptide for all vertebrates including mammals (Harder et al., 2001a). The widespread distribution of TRH and TRH receptors in several non-neuronal tissues suggests additional important functions for this neuropeptide, well beyond its classical role within the hypothalamic-pituitary-thyroid axis (Gary et al., 2003). In fact, TRH has been found to act as a multifunctional hypophysiotropic factor in vertebrates (Galas et al., 2009).

TRH signals are transmitted across the plasma membrane by TRH receptors, which are instrumental in converting extracellular ligand binding into intracellular signaling events (Engel and Gershengorn, 2007). TRH receptors belong to the superfamily of receptors coupled to GTP-binding proteins (GPCRs), the largest group of transmembrane spanning proteins in the vertebrate genome. These receptors are involved in a range of signaling pathways regulated by G proteins, including phosphoinositide-specific phospholipase C (PLC), adenylyl cyclase (AC), mitogen-activated protein kinase (MAPK), and calcium/calmodulin-dependent protein kinase (CAMK) (Sun et al., 2003). The involvement of certain intracellular signaling cascades triggered by TRH receptors may depend on the concrete experimental conditions. Among other molecules also β-arrestins as key regulators of GPCR signaling and trafficking can distinctively participate in both TRH receptor-initiated signal transmission and desensitization.

This review will summarize current knowledge about TRH receptors and the molecular basis of signaling processes regulated by these receptors. Special attention will be focused on potential cytoprotective and neuroprotective effects of TRH and its analogs and the molecular mechanisms underlying these beneficial effects. Understanding the physiological role of TRH receptors and uncovering the molecular mechanisms of signal transduction processes regulated by these receptors represent an important basis for specific and effective pharmacological modulation of this complex signaling system.

## Thyrotropin-releasing hormone receptors

Thyrotropin-releasing hormone receptors are divided into three subtypes, TRH-R1, TRH-R2, and TRH-R3. In humans, there is only one type of TRH-R, which is designated TRH-R1. Several other species of mammals including rodents express a second type of the receptor, TRH-R2. TRH-R3 is together with TRH-R1 expressed in birds. Despite the fact that both TRH-R1 and TRH-R2 stimulate the same signaling pathway, they are distributed differently in the brain and peripheral tissues (Sun et al., 2003). In general, TRH-R1 is thought to be mainly involved in regulating neuroendocrine responses, whereas TRH-R2 mainly mediates neurotransmitter effects (Monga et al., 2008). Thus, these receptor subtypes might have distinct biological functions. However, a study by Thirunarayanan et al. (2013) revealed that the TRH-R agonist taltirelin exerts its effects in the mouse central nervous system primarily through TRH-R1. These data suggest that the specific involvement of TRH-R subtypes is likely to be highly dependent on the type of agonist and the experimental model. Previous studies have shown that TRH-R1 and TRH-R2 have similar binding affinity for TRH and both stimulate the phosphoinositide/calcium signaling pathway. By way of comparison with TRH-R1, TRH-R2 was found to be more rapidly internalized and greatly downregulated after TRH binding (O’Dowd et al., 2000). Additionally, TRH-R2 exhibits a higher basal (ligand-independent) signaling activity than TRH-R1 (Wang and Gershengorn, 1999). The function and tissue distribution of TRH-R3 was recently investigated in chicken (Li et al., 2020). The tissue distribution and amino acid sequences of TRH-R1, TRH-R2, and TRH-R3 were previously compared in a teleost fish, medaka. The nucleotide and amino acid sequences of the different subtypes of TRH-R in medaka are 44%–60% and 48%–66% identical, respectively (Mekuchi et al., 2011).

### Structure and activation of the thyrotropin-releasing hormone receptor

**FIGURE 1:**
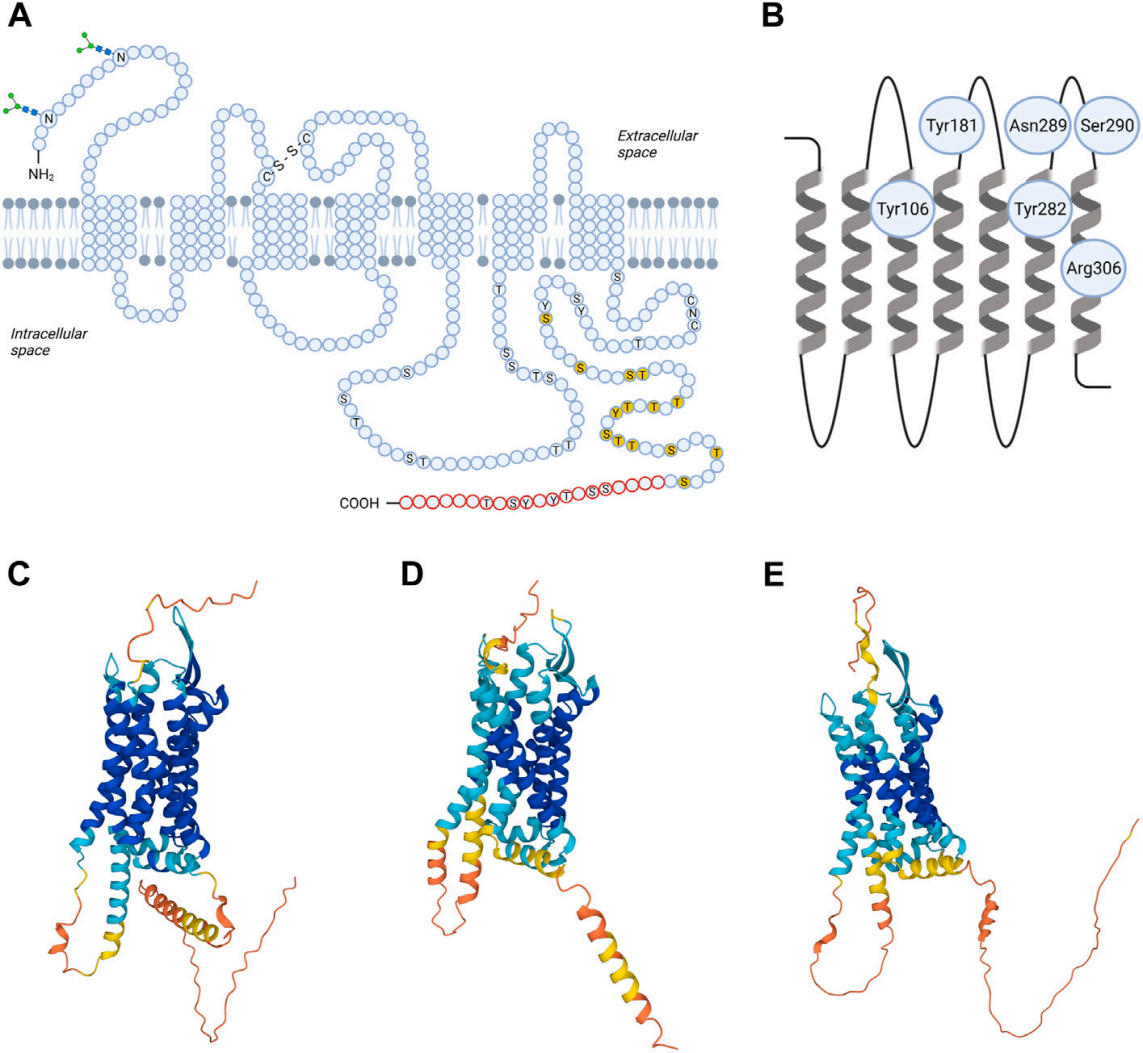
Structural models of TRH receptor. Shown is schematic two-dimensional topology **(A)** and ribbon model of rat TRH-R1 with amino acid residues possibly involved in TRH binding **(B)** and three-dimensional (3D) models of TRH-R1 **(C)**, TRH-R2 **(D)** and TRH-R3 **(E)**. TRH-R is an integral membrane protein containing seven membrane-spanning helices with an extracellular N-terminus and an intracellular C-terminus. The transmembrane helices are connected by three extracellular and three intracellular loops. Asn3 and Asn10 are glycosylated (“sugar trees” on the N-terminus). Cys98 in the first and Cys179 in the second extracellular loop are linked by a disulphide bond. Twenty C-terminal amino acids (in red) are missing in the short form of TRH-R. Residues 335-337 (CNC) and phosphorylatable residues in the C-terminal region and IC3 loop are denoted by the one-letter code; those already shown to by critical for desensitization/internalization of the receptor are highlighted in yellow. C, Cys; N, Asn; S, Ser; T, Thre; Y, Tyr; -S-S- disulphide interaction. 3D structural models for rat TRH-R1 with UniProt ID Q01717 **(C)**, rat TRH-R2 with UniProt ID Q9QWW3 **(D)**, and chicken TRH-R3 with UniProt ID A0A4P9IVJ2 **(E)** were provided by the AlphaFold Database (https://alphafold.ebi.ac.uk). The color scheme represents the model confidence. AlphaFold produces a per-residue confidence score (pLDDT) between 0 and 100. The colors dark blue, light blue, yellow, and orange represent very high (pLDDT > 90), medium (90 > pLDDT > 70), low (70 > pLDDT > 50), and very low (pLDDT < 50) confidence, respectively. Some regions below 50 pLDDT may be unstructured in isolation. The figure was created by BioRender.

TRH-R is a member of the rhodopsin/β-adrenergic receptor subfamily of GPCRs (Kakarala and Jamil, 2014). The cDNA encoding pituitary TRH-R was originally isolated from mice (Straub et al., 1990). Then, human TRH-R was cloned (Duthie et al., 1993). Although the crystal structure of TRH-R has not yet been solved, its spatial organization is predictable. It has an extracellular amino terminus, typical seven transmembrane domains (TM), three extracellular loops (EL1, 2, and 3) with several extracellular glycosylation sites and disulphide bond between the first and second EL, three intracellular loops (IL1, 2 and 3), and a cytoplasmic carboxyl tail. Schematic amino acid sequence structure and topology of TRH-R is depicted in Figure 1. Analysis of TRH-R expression in GH3 cells revealed two molecularly distinct receptor splice variants which differ in the presence or absence of a 52-amino acid segment in the C-terminal tail of TRH-R but not in the signaling characteristics (de la Peña et al., 1992). Upon TRH binding, both short and long isoforms of TRH-R stimulate formation of IP_3_ through G_q/11_α protein and release of Ca^2+^ from intracellular stores (Lee et al., 1995).

Recently, the 3D structural models for TRH-R subtypes were predicted from amino acid sequences and provided in the AlphaFold database (Jumper et al., 2021; Varadi et al., 2021). Comparison of the 3D structures of rat TRH-R1 (Figure 1C), rat TRH-R2 (Figure 1D), and chicken TRH-R3 (Figure 1E) revealed several differences in IL3, the extracellular N-terminus, and the cytoplasmic C-terminal tail. First, the α-helical structure of the transmembrane domain TM5 in rat TRH-R2 continues to the edge of IL3, resulting in IL3 being shorter than in rat TRH-R1 and chicken TRH-R3. Second, the N-terminal and C-terminal tails of rat TRH-R2 are shorter than those of rat TRH-R1 and chicken TRH-R3. Third, the N-terminal tails of rat TRH-R2 and chicken TRH-R3 are more structured near the TM1 than those of rat TRH-R1. Compared with chicken TRH-R3, the C-terminal tails of both rat TRH-R subtypes contain long α-helices that have different orientations due to different lengths and structures of the turns at the beginning of the C-terminal tail.

The extracellular loops of TRH-R are fundamental for the initial interactions with a ligand. It has been proposed that Asn289 and Ser290 of EL3 of rat TRH-R? ensure hydrogen bond interactions with the N-terminal part of the TRH molecule (Han and Tashjian, 1995a). However, Tyr188, Tyr192, Phe196, and Phe199 from EL2 and the upper part of the fifth transmembrane helix of TRH-R? cloned from GH_4_C_1_ rat pituitary cells are also important for interaction with the histidine residue of TRH (Han and Tashjian, 1995b). Besides that, direct binding contacts between pyroGlu of TRH and two residues (Tyr106 and Asn110) in TM3 of mouse TRH-R? were described (Perlman et al., 1994a; Perlman et al., 1994b). Subsequently, two additional direct contact sites between TRH and mouse TRH-R? were revealed: interaction between Tyr282 in TM6 and His of TRH, and interaction between Arg306 in TM7 and ProNH_2_ of the ligand (Perlman et al., 1996). Molecular models of the mouse TRH-R? binding pocket were used to describe known interactions but also to identify other groups that are potentially involved in binding the ligand (Laakkonen et al., 1996). Residues that bind TRH form “entry channel” to the TM binding pocket (Perlman et al., 1997). Interestingly, it was shown that the disulphide interaction between extracellular cysteines Cys98 in EL1 and Cys179 in EL2 of rat and mouse TRH-R? is not involved in receptor activation but is essential for preservation of the correct and high affinity conformation of the receptor (Perlman et al., 1995; Cook et al., 1996). Despite the fact that the conformational states of TRH-R1 and TRH-R2 are similar after TRH binding, surprisingly, optimized models of mouse TRH-R indicate that Trp279 mediates the direct interaction between TRH and TRH-R2 but not TRH-R1 (Deflorian et al., 2008). Recently, TRH and its analog taltirelin were reported to differ in their interactions with some residues of the human TRH-R binding pocket (Yang et al., 2022). According to this study, the hydrogen bonds linking TRH to Tyr192 and Asn289 from the TRH-R binding pocket are stronger and the binding pocket is narrower than in the case of taltirelin. Upon binding of TRH or taltirelin, different conformational changes also occur in TM6 and TM7 (Yang et al., 2022). These structural changes may be responsible for the lower signaling potency but higher intrinsic efficacy of taltirelin compared to TRH, as taltirelin acts as a superagonist at human TRH-R (Thirunarayanan et al., 2012).

It is known that the C-terminal tail of TRH-R plays an important role in interactions with β-arrestin as well as in the process of receptor internalization. β-Arrestin binds to phosphorylated sites in the C-terminus of TRH-R. Receptor desensitization and internalization seem to be dependent on phosphorylation of specific phosphosites in the receptor tail. A study from 1993 demonstrated that amino acids (AA) 335-337 in the C-terminus of mouse TRH-R are important for ligand driven receptor internalization (Nussenzveig et al., 1993a). The same AA sequence also appears to be important for sequestration of functionally uncoupled TRH-R and to play a role in the prevention of constitutive internalization (Petrou et al., 1997). In 2001, the C-terminal tail of rat TRH-R1 was studied in detail to determine which parts of the C-terminus are involved in these processes (Drmota and Milligan, 2000; Groarke et al., 2001). Jones and coworkers defined a region in the carboxyl tail of TRH-R1 from GH3 cells that binds β-arrestin in response to binding of an agonist that is critical for desensitization and internalization of the TRH receptor (Jones et al., 2007). TRH stimulates phosphorylation between AA 355-365 and 371-391, and phosphorylation of Thr365 was found to be critical for β-arrestin recruitment and subsequent desensitization and internalization of rat TRH-R1 (Jones and Hinkle, 2008). The rate of phosphorylation and dephosphorylation was found to depend not on the C-terminal sequence of the TRH-R1 but rather on regions outside the cytoplasmic tail (Gehret and Hinkle, 2010).

Because mutations in some GPCRs have been shown to be involved in pathological conditions in humans (Fukami et al., 2018), TRH-R was examined for the presence of mutations in various pituitary adenomas (Dong et al., 1996; Faccenda et al., 1996). Surprisingly, an intact DNA coding sequence of TRH-R was detected in all human pituitary adenomas examined (Dong et al., 1996; Faccenda et al., 1996), making it possible that some other components of the TRH-R signaling pathway are involved in pituitary tumorigenesis. In contrast, two different inherited mutations in the TRH-R gene were found in a patient suffering from hypothyroidism, resulting in decreased or even absent biological function were found in a patient suffering from hypothyroidism (Collu et al., 1997). One of the mutations was located at position 49, resulting in replacement of arginine (CGA) with a stop codon (TGA) at peptide position 17 in the maternal allele. A mutation of the paternal allele consists of a deletion of nucleotide positions 343 to 351 and the replacement of alanine (GCC) by threonine (ACC) at position 352 (Collu et al., 1997). There are other examples of mutant TRH-R associated with hypothyroidism (Bonomi et al., 2009; Koulouri et al., 2016; García et al., 2017). Another patient (Collu et al., 1997) was diagnosed with a homozygous nonsense mutation at position 17 (R17X) similar to that mentioned above (Bonomi et al., 2009). A point mutation at proline 81 (P81R) in TM2 of TRH-R or an I131T mutation at a highly conserved hydrophobic position in IL2 have also been associated in hypothyroidism (Koulouri et al., 2016).

GPCR-mediated signaling has been shown to be modulated by posttranslational modifications of a receptor, such as glycosylation, phosphorylation, and palmitoylation (Patwardhan et al., 2021). No detailed information is available on TRH-R glycosylation, but phosphorylation of this receptor has been studied quite extensively. It was found that dimerization of rat TRH-R1 enhances agonist-dependent receptor phosphorylation (Song et al., 2007) and that phosphorylation of the rat TRH-R plays an important role in subcellular trafficking of the receptor (Jones and Hinkle, 2009). A study examining the role of cysteine residues on the carboxyl tail of mouse TRH-R1 and their palmitoylation revealed that a single palmitoylation site in the proximal carboxyl tail is sufficient to constrain the receptor in an inactive conformation (Du et al., 2005). Interestingly, the long isoform of rat TRH-R can be posttranslationally modified by ubiquitination, which allows degradation of misfolded, newly synthesized receptors but is not required for proper signal transduction or receptor internalization (Cook et al., 2003).

### Thyrotropin-releasing hormone receptor desensitization and internalization

**FIGURE 2:**
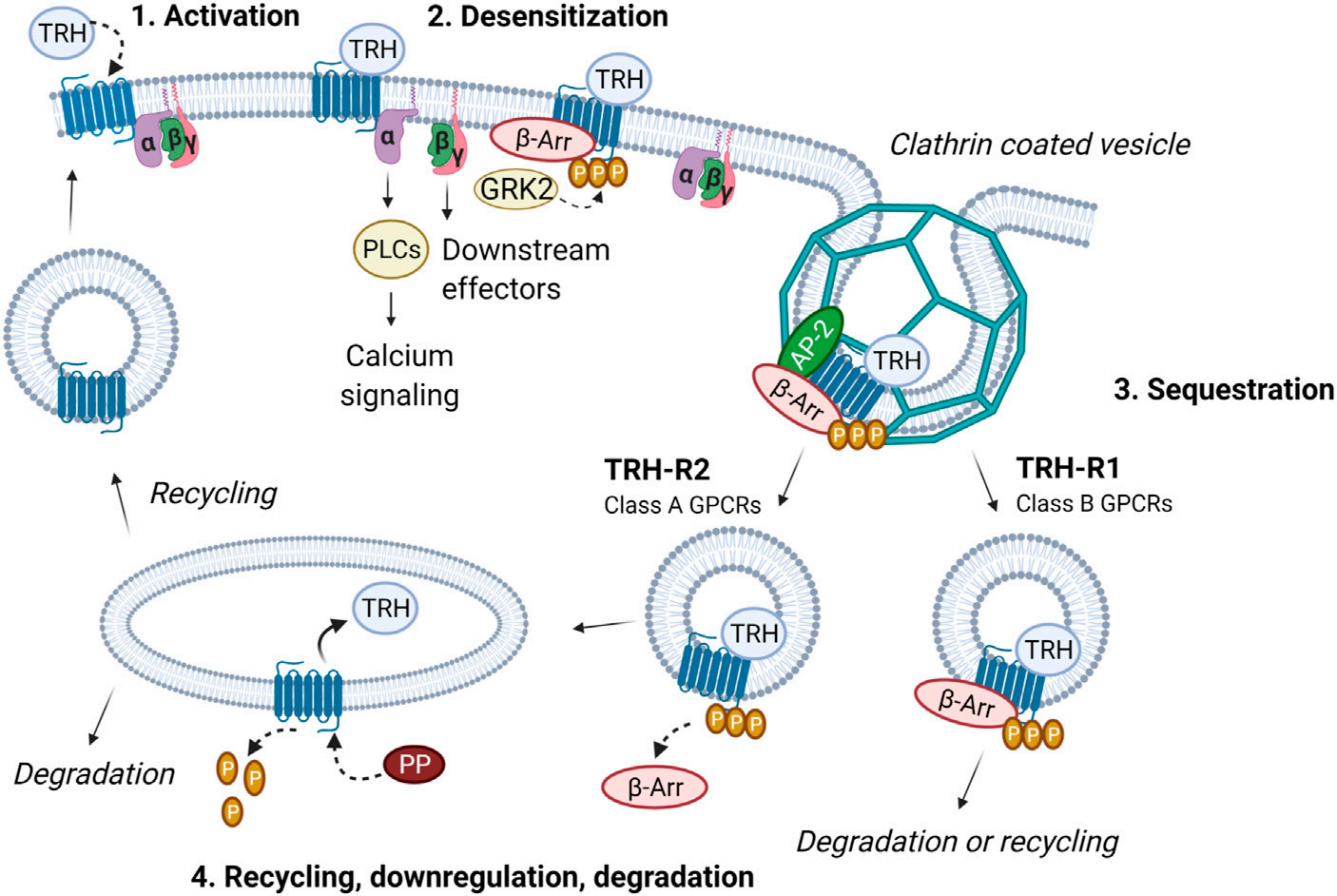
Intracellular trafficking of TRH receptor subtypes. The activation of TRH-R with TRH results in dissociation of Gα and Gβγ and an increase in intracellular calcium. Receptor is then desensitized through phosphorylation by GRK2 and β-arrestin binding. In this way, coupling between the receptor, G protein and effector is abolished and signaling terminated. β-Arrestin functions as an adaptor protein which recruits proteins from the endocytic machinery (i.e., clathrin, AP-2 and others). Subsequently, receptor is sequestered *via* clathrin-coated vesicles. Internalized TRH-R can then undergo two different processes depending on the receptor subtype and interaction with β-arrestin. TRH-R2, belonging to class A receptors, dissociates from β-arrestin immediately upon internalization. TRH is removed and receptor is dephosphorylated by protein phosphatase (PP). TRH-R2 is then recycled and targeted to the plasma membrane. On the other hand, TRH-R1 which belongs to the class B receptors, forms stable complexes with β-arrestin in endocytic vesicles and can either be degraded or slowly recycled to the plasma membrane. The figure was created by BioRender.

Early on, TRH-R, similar to other GPCRs, was found to be rapidly phosphorylated and desensitized after binding of an agonist (Anderson et al., 1995; Jones et al., 2007). Phosphorylation is carried out by a family of Ser/Thr protein kinases, the G protein-coupled receptor kinases (GRKs). In the case of rat TRH-R, it is mainly GRK2 that binds exclusively to and inhibits G_q/11_-coupled receptors (Heximer et al., 1997). The interaction of GRK2 with the G_q_α subunit can be enhanced by tyrosine phosphorylation by c-Src kinase (Mariggio et al., 2006). However, GPCRs can also be phosphorylated by other kinases such as protein kinase (PKA) or protein kinase C (PKC). While there is no information on the involvement of PKA in the desensitization of TRH-R, the possible role of PKC in the desensitization of the receptor was described by Grimberg et al. (1999) in AtT20 pituitary cells. The phosphorylated receptor attracts β-arrestin to the plasma membrane, where it disrupts interaction between the receptor and its cognate G protein, thereby terminating signal transduction. TRH-R with the bound agonist is then internalized along with β-arrestin *via* clathrin-coated vesicles and either recycled or degraded as mentioned above (Figure 2). It has been found that internalized mouse TRH-R? can return to the membrane within 20 min after removal of the agonist (Ashworth et al., 1995). Interestingly, a study performed with rat TRH-R fused to a Timer protein showed that a substantial fraction of internalized TRH-R2 is not recycled even after several hours, but rather the plasma membrane is replenished with new receptors from the intracellular pool (Cook and Hinkle, 2004b). This finding was basically confirmed by further experiments on different model cells (Jones and Hinkle, 2009). Prolonged agonist treatment may lead to subcellular redistribution not only of TRH-R but also of its cognate G proteins. However, rat TRH-R1 and G_q/11_α are internalized and redistributed on different time scales in response to agonist stimulation (Drmota et al., 1998a; Drmota et al., 1999). In contrast to the long isoform of rat TRH-R1, internalization, trafficking, and downregulation proceed much slower for the G_q/11_α subunits (Drmota et al., 1998b). It was also observed that both G_q_α and G_11_α are comparably downregulated after cell exposure to TRH (Kim et al., 1994). Another study demonstrated that not only Gα but also Gβ proteins were redistributed and downregulated in response to TRH (Svoboda et al., 1996). Interestingly, prolonged treatment of cells with TRH led to marked translocation of G_q/11_α from detergent-insensitive membrane domains to the bulk membrane phase (Pesanova et al., 1999), although rat TRH-R1 is predominantly not localized in lipid rafts (Rudajev et al., 2005). The possible role of membrane rafts in TRH-R-mediated signaling remains unclear.

The molecular mechanisms underlying desensitization, trafficking, and resensitization of TRH-R have been the subject of intense investigation, particularly in the late 1990s and early 21st century (Drmota et al., 1998a; Yu and Hinkle, 1998; Zaltsman et al., 2000; Cook and Hinkle, 2004b; Jones and Hinkle, 2009). It was found that stimulation by agonists results in internalization of rat TRH-R *via* clathrin-coated pits and that, after endocytosis, vesicles containing phosphorylated receptors fuse with rab5-positive vesicles, which form early sorting endosomes rich in rab5 and rab4. Interestingly, dephosphorylated TRH-Rs accumulate in rab4-positive, rab5-negative recycling endosomes, but the mechanisms responsible for this sorting are not yet known. It appears that protein phosphatase 1 is involved in dephosphorylation of the receptor but the details of how the enzyme is targeted to the receptor remain unclear (Hinkle et al., 2012). There is some evidence that phosphorylation/dephosphorylation of proteins interacting with Rab4 or Rab5, depending on the amount of cellular β-arrestin2, may underlie, at least in part, the development of endocytic processes triggered by agonist stimulation of rat TRH-R (Drastichova et al., 2022).

In general, TRH-R-mediated signaling is markedly desensitized after TRH treatment. Importantly, the IP_3_ response to TRH shows homologous desensitization, whereas the Ca^2+^ response shows heterologous desensitization. This can be explained by the fact that depletion of intracellular Ca^2+^ pools prevents the response to other stimuli (Yu and Hinkle, 1998). It has been observed that the magnitude of the Ca^2+^ response depends not only on the expression level of the receptor but also on G_q/11_α, which may strongly influence the process of desensitization (Novotny et al., 1999; Ostasov et al., 2008). Interestingly, significantly lower sensitivity to TRH stimulation was observed in cells after cholesterol depletion, suggesting that the intact structure of plasma membranes is crucial for functional coupling between the long isoform of rat TRH-R1 and G_q/11_α (Ostasov et al., 2007; Brejchova et al., 2015).

Extremely important molecules that modulate signal transduction and trafficking of many GPCRs and are required for receptor internalization, are β-arrestins. β-Arrestins are cytosolic proteins that were originally identified as proteins that stop signal transduction by binding to receptors. It is now clear that the role of β-arrestins is more complex, as they can also regulate GPCR trafficking and cellular signal transduction. There are two subtypes of β-arrestins, β-arrestin1 and β-arrestin2, which are ubiquitously expressed (Tian et al., 2014). After recruitment to the phosphorylated receptor, they serve as adaptors to link the receptor to components of the endocytic machinery, such as clathrin and adaptor protein 2 (AP-2). The amount of cellular β-arrestin2 appears to be a key factor determining the phosphorylation pattern of the AP-2 *α* subunit or some proteins interacting with the AP-2 complex (Drastichova et al., 2022). β-Arrestins also function as scaffolding signaling adaptors. They can recruit Src kinases to receptors (Luttrell et al., 1999) and assemble components of MAPK cascades such as Raf-1, MEK1, and ERK1/2 to promote signal transduction (Luttrell et al., 2001; Coffa et al., 2011; Cassier et al., 2017). They can bind a wide range of other proteins and are translocated to the nucleus, where they regulate gene expression (Kang et al., 2005; Rosano et al., 2013). β-Arrestins are critical for regulating TRH-R function and play an important role in receptor desensitization (Groarke et al., 2001; Jones and Hinkle, 2005). The intracellular C-tail domain of rat TRH-R has been identified as important for β-arrestin-dependent receptor internalization (Hanyaloglu et al., 2001). Interestingly, rat TRH-R subtypes have been shown to be differentially regulated by β-arrestin1 and β-arrestin2 and the interactions of TRH-R with β-arrestin can be altered by the formation of receptor hetero-oligomers (Hanyaloglu et al., 2002).

Experiments on mouse embryonic fibroblasts lacking G_q/11_α have shown that these G proteins are not necessary for agonist-induced mouse TRH-R internalization but are required for triggering Ca^2+^ response (Yu and Hinkle, 1999). In addition, these authors used a truncated TRH receptor lacking potential phosphorylation sites in the cytoplasmic C-terminus, and they found that such a receptor is able to transduce signals but does not internalize or cause membrane localization of β-arrestin (Yu and Hinkle, 1999). These results demonstrate that calcium signaling by mouse TRH-R requires coupling to G_q/11_α, but TRH-dependent binding of β-arrestin and sequestration do not.

Rather unexpectedly, it was observed in two independent studies that prolonged treatment of cells with TRH can lead to an upregulation of the number of rat TRH-R1. A significant increase in receptor protein and its subcellular redistribution was detectable after only 5 h (Drmota et al., 1999), and it was more pronounced after 48 h of treatment (Cook and Hinkle, 2004a). The increased TRH-R1 expression could only be partially attributed to changes in receptor mRNA, which increased only slightly. TRH-R1 upregulation was mimicked by phorbol ester and blocked by agents that inhibit PKC and the calcium response, and the number of TRH-R1 was slowly reversible after hormone withdrawal. It appears that TRH increases the number of receptors by a complex mechanism that requires signal transduction but not endocytosis of the receptors (Cook and Hinkle, 2004a). It was also found that long-term treatment of cells with TRH can alter the expression of several proteins (Drastichova et al., 2010). A similar finding was noted previously using gene array technology in pancreatic beta cells, where TRH significantly stimulated several groups of genes, including GPCRs, cell cycle regulators, protein turnover factors, growth factors and those involved in insulin secretion, endoplasmic reticulum traffic mechanisms, and DNA recombination (Luo and Yano, 2005). Thus, prolonged activation of TRH-R can have a number of consequences, in addition to desensitization of the receptor.

### Molecular complexes of the thyrotropin-releasing hormone receptor

Homo- and heterodimerization of GPCRs or tight interactions with other cellular proteins is a common phenomenon that has important implications for the regulation of cellular processes (Dijkman et al., 2018). Many types of receptors occur as dimers because in this state they possess high-affinity binding sites for interaction with ligands. It has been observed that TRH receptors can form homo- and hetero-oligomers in living cells. The formation of TRH-R1–TRH-R2 hetero-oligomers may affect the kinetics of receptor internalization. Rat TRH-R2 has been found to be internalized more slowly than TRH-R1 (Hanyaloglu et al., 2002). TRH-R subtypes are also divided into two classes depending on the type of β-arrestin they preferentially use for internalization and whether or not they internalize with bound β-arrestin (Figure 2). TRH-R1, which belongs to class B receptors, uses both β-arrestins equally and internalizes with β-arrestin *via* clathrin-coated vesicles. TRH-R2 binds preferentially to β-arrestin2, which dissociates from the receptor after its internalization, and thus belongs to the class A receptors (Hanyaloglu et al., 2002). Homo- and hetero-oligomerization of TRH-Rs can affect their trafficking and represents a possible mechanism for the differential cellular responses elicited by agonist stimulation. The differences in signaling and internalization of TRH-R1 and TRH-R2 may be important for the engagement of these receptor subtypes in mammalian physiology (O’Dowd et al., 2000).

The existence of homomeric TRH-R complexes was first demonstrated by bioluminescence resonance energy transfer (BRET) method using the construct of rat TRH-R1 with bioluminescent enzyme (Kroeger et al., 2001). This study, which focused on regulated dimerization of the rat TRH-R1, revealed that dimerization of this receptor is probably insufficient to stimulate downstream signaling pathways. On the other hand, dimeric forms of TRH-R1 may play a role in agonist-induced receptor internalization and endocytosis (Song and Hinkle, 2005). It has been reported that stimulation with TRH increases the formation of rat TRH-R1 dimers (Zhu et al., 2002). Dimerization of rat TRH-R1 enhances its phosphorylation, and receptor dimerization may therefore have a significant effect on the efficacy and duration of signal transduction (Song et al., 2007).

In 1993, it was proposed that TRH-R can form complexes with agonists, G proteins, and probably phospholipase C after agonist stimulation by an agonist using mouse pituitary TRH-R (Nussenzveig et al., 1993b). Subsequently, Petrou and Tashjian (1998) investigated the hypothesis that G_q_α and G_11_α need not dissociate from receptors during internalization at the plasma membrane, and they confirmed that TRH-R and the G_11_α subunit are internalized together in clathrin-coated vesicles in GH_4_C_1_ cells. Later studies based on the use of clear native polyacrylamide gel electrophoresis revealed the presence of preassembled complexes of rat TRH-R1 and G_q/11_α (Drastichova and Novotny, 2012a). Treatment with TRH resulted in the dissociation of high-molecular-weight complexes of G_q/11_α, the formation of low-molecular-weight complexes containing G_q/11_α, and the concomitant translocation of this protein to the cytosol (Drastichova and Novotny, 2012b). In this context, it is worth noting that TRH receptors can signal persistently under appropriate conditions and that sustained signaling correlates with G protein and receptor levels. It has been suggested that persistent signaling by TRH receptors occurs when sufficient levels of agonist/receptor/G protein complexes are present and that TRH-R2 forms and maintains these complexes more efficiently than TRH-R1 (Boutin et al., 2012). It is reasonable to speculate that the formation of complexes of TRH-R, G proteins, and possibly other components such as G protein regulatory proteins and adaptors may be important for receptor-mediated signal transduction in both conventional and unconventional ways. It has been shown that the presence or absence of different interacting partners of human TRH-R can strongly influence the mobility of the receptor in the plasma membrane, further supporting the notion of the existence of receptor complexes (Moravcova et al., 2018). Further studies are needed to elucidate the role of TRH-R complexes and other participants in TRH receptor-mediated signaling.

## Thyrotropin-releasing hormone receptor-mediated signaling

**FIGURE 3:**
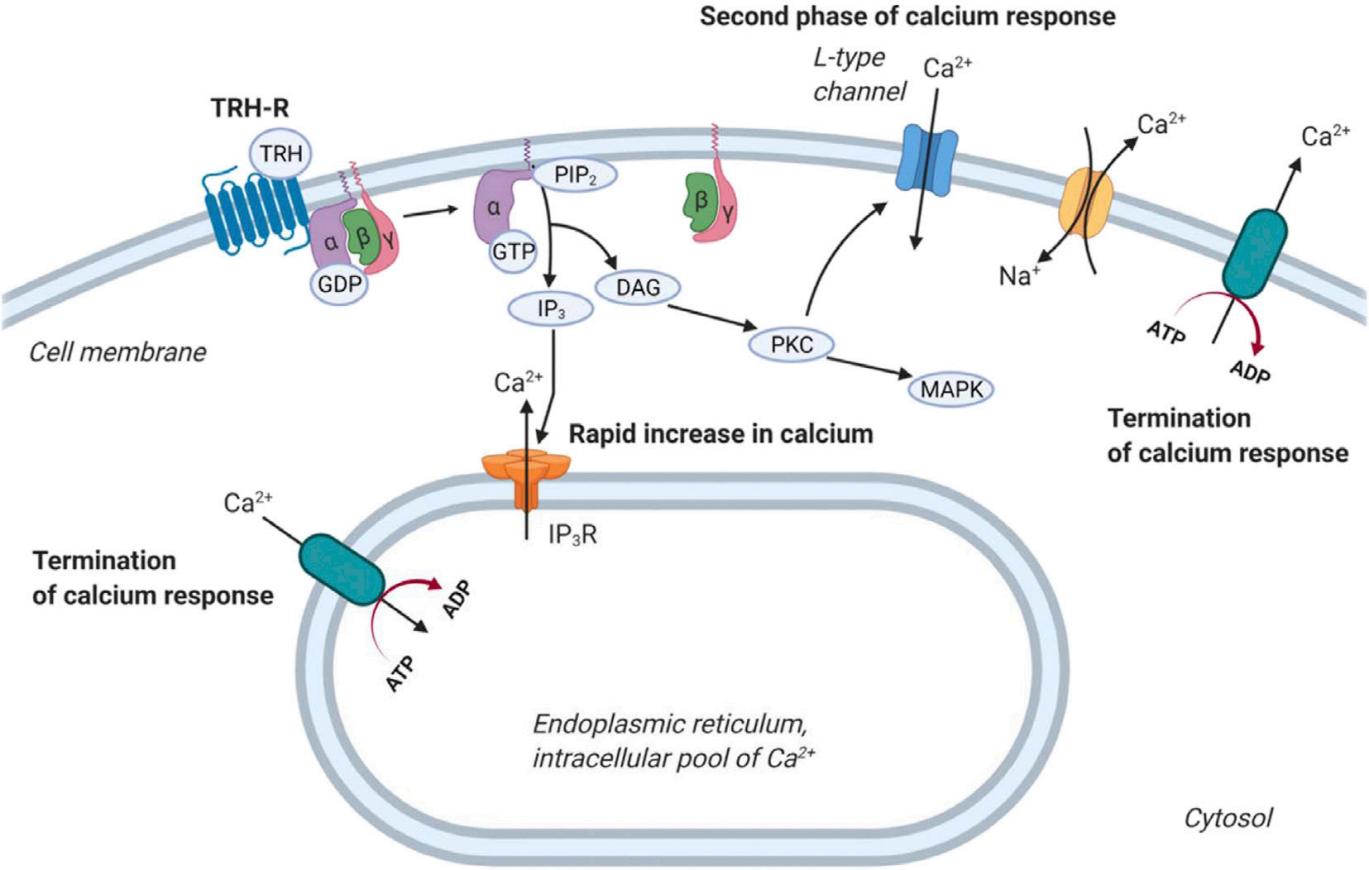
Regulation of calcium release by TRH receptor. At first, TRH acting through the G protein coupled TRH-R activates the cleavage of PIP_2_ into IP_3_ and DAG. IP_3_ then triggers calcium release from intracellular stores 1). After that, calcium pumps drive Ca^2+^ away from the cytosol 2). Plasma membrane-bound calcium channels are responsible for the second phase of calcium release from the extracellular space 3). Na^+^/Ca^2+^ exchanger removes calcium from cells. The figure was created by BioRender.

TRH exerts its biological responses *via* TRH-R, a transmembrane receptor found predominantly in the thyrotropic cells of the anterior pituitary, but has also been detected in other cell types (Gershengorn and Osman, 1996; Mellado et al., 1999; Minakhina et al., 2020). TRH-R is classically coupled to G_q/11_ proteins, and its activation by an agonist leads to stimulation of PLCβ. This enzyme catalyzes the hydrolysis of phosphatidylinositol-4,5-bisphosphate (PI(4,5)P_2_) to IP_3_ and DAG. These 2 s messengers initiate the mobilization of calcium from the cell’s internal stores and the activation of PKC, respectively. Intracellular calcium and PKC then trigger a number of cellular responses, including activation of MAP kinase signaling cascades or regulation of ion channels (Hinkle et al., 2012; Kanasaki et al., 2015; Joseph-Bravo et al., 2016). Interestingly, activation of TRH-R can control intracellular Ca^2+^ concentration in at least three different ways (Figure 3). First, it mobilizes IP_3_-sensitive intracellular calcium stores, resulting in an initial rapid increase in intracellular Ca^2+^ concentration (Nelson and Hinkle, 1994a). Second, it stimulates the Ca^2+^ pump, which helps to terminate the initial Ca^2+^ spike and shunts calcium ions out of the cytosol (Nelson and Hinkle, 1994b). Finally, activation of TRH-R may also affect the influx of Ca^2+^ from the extracellular environment that is responsible for the sustained second phase of the Ca^2+^ response, which appears to be mediated by PLC (Gollasch et al., 1993; Nelson and Hinkle, 1994a). From a recent study, the main source of cytosolic Ca^2+^ after TRH treatment is the endoplasmic reticulum (Rojo-Ruiz et al., 2021).

Nevertheless, TRH-R turned out to be able to activate more than one second messenger system under certain circumstances. In addition to the G_q/11_-regulated PLC pathway, TRH-R may also activate AC through direct interaction with the stimulatory G_s_ protein (Paulssen et al., 1992a; Johansen et al., 2001b). In addition, there is some evidence from experiments with GH3 cells that TRH-R can couple to the inhibitory G_i_ proteins that do not cause activation of AC (Paulssen et al., 1992a). Another study found that G_s_α in *Xenopus* oocytes can most likely couple the long isoform of rat TRH-R to PLC activation and subsequent IP_3_ formation and Ca^2+^-mediated response (de la Peña et al., 1995). Prolactin release has been reported to be driven by TRH-R, which is functionally linked to G_s_α in lactotrophs (Kineman et al., 1996), and the same interaction was found in GH_4_C_1_ rat pituitary tumor cells (Gordeladze et al., 1988). In addition to its direct stimulatory effects on G_s_, TRH stimulated the formation of 3′,5′cyclic adenosine monophosphate (cAMP) in GH_4_C_1_ prolactin-producing cells, thereby activating cAMP-dependent PKA (Gautvik et al., 1977). The effects of TRH on the increase in activity of AC were also found in the anterior pituitary of the rat (Brozmanova et al., 1980). Furthermore, there are some data from GH3 cells suggesting that TRH and other hormones from the hypothalamus likely modulate levels of G proteins, thereby favoring appropriate signaling (Paulssen et al., 1992b; Paulssen R. H. et al., 1992). G_13_α and Gβγ have also been shown to contribute importantly to TRH-R-mediated effects in GH3 cells (Miranda et al., 2005). The interplay of second messenger systems and cross-talk between hormone signaling systems is of great importance in the cellular response to extracellular signals. The effects of TRH on AC or even cyclic nucleotide phosphodiesterases remain controversial, as some studies refuted these effects (Hinkle and Tashjian, 1977) and there are no recent studies addressing this question. Activated TRH-R is likely to trigger different effector systems that may elicit different responses in different cells. The specific experimental conditions and the type of cells/tissues are likely to be the most important factors determining the specific molecular mechanism involved in TRH receptor-mediated signaling. Further studies of post-receptor signaling mechanisms are desirable to unravel the details at the molecular level.

### Regulation of thyrotropin-releasing hormone receptor-mediated signaling

The process of signal transduction from GPCRs to their cognate G proteins and downstream targets is modulated by a number of different factors. Among them, plasma membrane composition, various posttranslational modifications, and interactions with regulatory proteins are the most important and most frequently studied. As mentioned above, the engagement of specific intracellular pathways and the outcome of TRH-R-mediated signaling may also depend on cell type.

There are four families of heterotrimeric G proteins classified by their *α* subunits (G_s_α, G_i/o_α, G_q/11_α, and G_12/13_α) that regulate specific downstream effectors and trigger specific pathways through a variety of membrane-bound receptors. Besides that, Gβγ dimers also contribute to the regulation of various effectors. Previous studies have shown that TRH-R is quite promiscuous, coupling predominantly to G_q/11_ but also interacting with G_s_ and G_i_ proteins in GH3 cells (Paulssen et al., 1992a; Johansen et al., 2001b; Boutin et al., 2012). Binding of a ligand to TRH-R results in an exchange of GDP for GTP at the Gα subunit. Subsequently, Gα dissociates from the Gβγ dimer and both interact with various downstream effectors such as PLCs, AC, phosphodiesterases, protein kinases, or ion channels that trigger various signaling pathways. The Gα subunit is then desensitized by its intrinsic GTPase activity, which inactivates its function and thus regulates the duration of the signal. The GTPase activity of the Gα subunit remains active for only a short period of time and is accelerated by regulators of G protein signaling (RGS) that function as GTPase activating proteins (GAPs) (Stewart and Fisher, 2015). Therefore, RGS proteins determine the extent and duration of cellular responses initiated by many GPCRs, including TRH-R. In addition to the 20 canonical mammalian RGS proteins that act as functional GAPs, there are nearly 20 other proteins that carry nonfunctional RGS homology domains, which often mediate interaction with GPCRs or Gα subunits. RGS4, which inhibits signaling through other receptors coupling to G_q/11_ proteins in different ways, was found to inhibit TRH-stimulated signaling through mouse TRH-R1 and TRH-R2 to a similar extent. Interestingly, other RGS proteins tested had no effect on TRH-R-mediated signaling (Harder et al., 2001b). Modulation of TRH-R-mediated signaling by RGS4 appears to be an important mechanism for regulating receptor activity.

G protein-coupled receptor kinases (GRKs) are other proteins that possess the RGS domain. There are seven GRKs (GRK 1-7) (Komolov and Benovic, 2018). GRKs are soluble proteins and therefore use different mechanisms to get close to the vicinity of membrane-bound GPCRs and G proteins. Some of them are prenylated, others are palmitoylated or are bound *via* Gβγ. These enzymes preferentially phosphorylate receptors occupied by an agonist. GRK2, a typical GPCR kinase, consists of 689 amino acid residues and three domain structures—N-terminal (catalytic) and C-terminal domains. It interacts exclusively with members of the G_q/11_α protein family. It binds directly to activated G_q_α subunits, inhibiting the activation of downstream effectors. In addition to its function in desensitizing receptors, GRK2 additionally regulate signal transduction by binding to the G_q_α subunit (Sallese et al., 2000). The binding site for interaction with G_q/11_α appears to be located in the amino-terminal domain of GRK2, the domain that shares homology with the RGS proteins. However, the actual binding site of GRK2 differs from the binding sites of other RGS proteins because it involves the COOH-terminus of its α5 helix and not other parts of the RH domain that other RGS proteins use (Sterne-Marr et al., 2003). Besides that GRK2 has been shown to discriminate between members of the G_q/11_α class (Day et al., 2003). And as mentioned above, c-Src-mediated phosphorylation of GRK2 enhances its interaction with G_q_α (Mariggio et al., 2006). GRK2 is one of the kinases that can be selectively regulated by free Gβγ subunits and membrane phospholipids. A thorough mutational analysis of the GRK2 PH domain revealed that the interaction between GRK2, Gβγ proteins, and negatively charged membrane phospholipids is essential for appropriate phosphorylation of the receptor. Experimental evidence suggests that downstream kinases such as PKC play at most a minor role in agonist-induced phosphorylation of TRH-R and suggests that receptor phosphorylation is primarily mediated by GRK2 (Jones and Hinkle, 2005; Jones et al., 2007; Gehret et al., 2010). As indicated above, GRK2-mediated phosphorylation of TRH-R is important not only for receptor desensitization but also for potential hetero-oligomerization of the receptor and thus may modulate specific cellular responses to agonist stimulation.

### Effectors of thyrotropin-releasing hormone receptor-mediated signaling

After activation of TRH-R, cognate G proteins and β-arrestins regulate the function of various effector molecules, which may lead to different cellular and physiological responses. Besides PLC and AC, TRH-R appears to be able to affect the function of MAPKs or phosphatidylinosil-3 kinase (PI3K)/Akt and Ca^2+^/calmodulin (CaM)-dependent kinases (CAMK). Activation of the PLC/PKC pathway and possibly the AC/PKA pathway by TRH-R has been frequently described in different animal and cellular models (Paulssen et al., 1992b; Nussenzveig et al., 1993b; Johansen et al., 2001a; Lei et al., 2001). Interestingly, both PKC and PKA appear to be independently critical for the effects on glycogen synthase kinase-3β (GSK-3β) mediated by TRH-R in rat hippocampal neurons (Luo and Stopa, 2004). Endocrine secretion of TSH in rat pituitary cells has been found to be controlled by CREB phosphorylation *via* the activity of CaMKII and CaMKIV, both of which are phosphorylated upon TRH stimulation (Altobelli et al., 2021). Much less is known about the involvement of other potential effectors of TRH-R.

**FIGURE 4:**
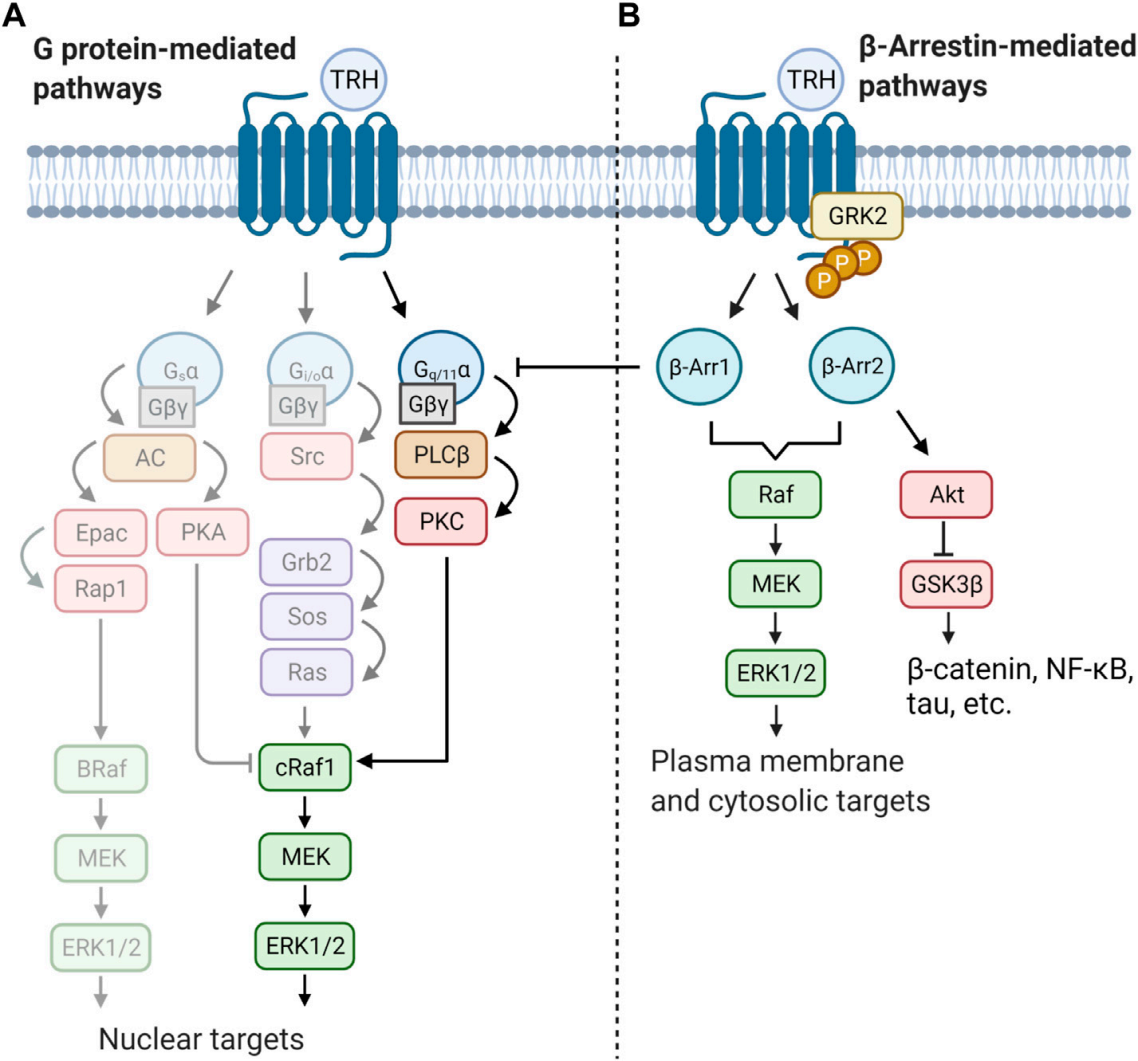
Schematic illustration of two distinct modes of regulation of MAPK cascades triggered by TRH receptor. Activity of ERK is controlled by both G protein and/or β-arrestin-mediated pathways. Activation of receptor molecule by an agonist can lead to different signalling outputs depending on the cell type and signalling molecule supply. Basically, **(A)** TRH signals through the canonical G_q/11_α–PLCβ–PKC pathway which can directly activate the first protein kinase from the MAPK cascade (cRaf1). Activated ERK then translocate to the cell nucleus. TRH may possibly activate other G proteins (lighter part) and further regulates gene transcription or cell cycle progression by phosphorylation of transcription factors. **(B)** β-Arrestin (β-Arr1 and β-Arr2) terminates G protein signaling by desensitization of TRH-R. Simultaneously it can function as scaffold activating another pool of ERK kinase which, afterwards, phosphorylates different substrates. The figure was created by BioRender.

Mitogen-activated protein kinases are evolutionarily conserved serine/threonine-specific protein kinases that are activated by a wide range of stimuli and regulate processes such as cell proliferation, cell differentiation, or apoptosis. The MAPKs are terminal kinases of cascades consisting of three sequentially activated protein kinases (Keshet and Seger, 2010). They can be divided into three major families in mammals: extracellular-signal-regulated kinases (ERKs), Jun amino-terminal kinases (JNKs), and stress-activated protein kinases (p38/SAPKs). The mode of activation of MAPKs depends on cell type and receptor equipment. Upon activation, G_q/11_α-coupled receptors trigger various signals involved in the control and regulation of all major members of the MAPK signaling pathways. To increase signaling efficiency between successive kinases in the cascade, increase signal fidelity by restricting cross-talk between two parallel kinase cascades, and target MAPKs to specific subcellular locations, MAPKs bind to scaffold proteins. One of the most representative scaffold molecule is β-arrestin, which can serve as a GPCR-regulated scaffold for MAPK activation. Interestingly, in addition to serving as downstream effectors, MAPKs can also act as negative regulators of GPCRs. Two phosphosites (Ser14 and Thr276) in β-arrestin were found to be critical for ERK1/2-triggered intracellular sequestration of the receptor (Paradis et al., 2015). Furthermore, it was shown that MAPK can phosphorylate a specific region in β-arrestin2 (Thr178) after internalization of the receptor complex of the receptor with β-arrestin2 in endosomes. This phosphorylation then drives signaling of the Raf-MEK-ERK1/2 cascade and plays a role in the endosomal trafficking and signaling of GPCRs (Khoury et al., 2014). There is some evidence that TRH-R may also affect the MAPK cascades, particularly the ERK/MAPK signaling pathway. TRH was observed to be able to stimulate MAPK activity in rat anterior pituitary cells in either a PKC-dependent or PKC-independent manner (Ohmichi et al., 1994a), and subsequent study unravelled the possible role of Gβγ in PKC-independent activation of ERK1/2 by TRH in COS-7 cells transfected with rat TRH-R (Palomero et al., 1998). TRH-induced activation of MAPK was shown to stimulate prolactin synthesis and secretion in GH3 cells (Kanda et al., 1994; Kanasaki et al., 1999). In the same cell model, TRH was found to affect the prolactin promoter through activation of PKC and influx of Ca^2+^ from L-type Ca^2+^ channels, leading to induction of MAPK (Wang and Maurer, 1999). Interestingly, TRH-R-mediated activation of the MAPK pathway in pituitary cells could be inhibited by dopamine D_2_ receptors through G_i3_ or G_o_ proteins, suggesting the ability of these proteins to inhibit MAPK through C-Raf and B-Raf-dependent inhibition of ERK1/2 kinase (Ohmichi et al., 1994b; Banihashemi and Albert, 2002). Smith et al. (2001) demonstrated, using mouse TRH-R that activation of ERK1/2 by TRH requires clathrin-dependent receptor endocytosis and PKC but is insensitive to pertussis toxin and does not require Ras or PI3K. Interestingly, TRH-induced tyrosine phosphorylation of the epidermal growth factor (EGF) receptor appears to be important for the complete activation of ERK1/2 by TRH in GH3 cells (Wang et al., 2000). These data support the notion of a close cooperation between TRH-R- and EGF receptor-mediated signaling systems. There is some evidence that PI3K/Akt may also be involved in TRH-R-mediated signaling (Jaworska-Feil et al., 2010; Sosa et al., 2012). However, the exact mechanism of how TRH-R may recruit the PI3K/Akt signalling pathway has not yet been described. Figure 4 summarizes several ways in which TRH-R may regulate MAPK signaling pathways *via* G proteins and β-arrestin.

There is ample evidence that TRH-R-mediated signaling is involved in the regulation of various ion channels. Besides triggering Ca^2+^ release from intracellular stores through IP_3_-sensitive Ca^2+^ channels, TRH-R may also affect several other ion channels. Interestingly, Gollash et al. reported that TRH-induced Ca^2+^ release from internal stores is followed by a phase of sustained Ca^2+^ influx through voltage-dependent Ca^2+^ channels stimulated by the concerted action of G_i2_ (and G_i3_) as well as PKC in GH3 cells (Gollasch et al., 1993). However, the major signaling component involved in the action of TRH-R on ion channels appears to be G_q/11_ proteins. Importantly, the effect of TRH-R agonists must be not only stimulatory but also inhibitory. TRH has been found to inhibit two-pore domain K^+^ channels *via* G_q/11_ proteins in rat hippocampus (Deng et al., 2006). TRH-mediated inhibition of the resting K^+^ conductance was independent of PLC, CAMKII or MAPK activity, suggesting direct coupling of G_q/11_α to potassium-selective leak channels. It was observed that TRH can also strongly inhibit G protein-coupled inwardly rectifying K^+^ (GIRK) channels and that this inhibition relies on G_q_α and PLC (Lei et al., 2001; Rojas et al., 2008). Several authors reported that activation of rat TRH-R may affect the function of voltage-activated, ether-a-go-go-related potassium channels (ERG), probably *via* ERK1/2 and ribosomal S6 kinase (RSK) and possibly Rho kinases (Barros et al., 1998; Storey et al., 2002; Carretero et al., 2012; Carretero et al., 2015). In this context, it is interesting to note that G_q/11_ protein does not appear to be involved in TRH-induced inhibition of endogenous ERG currents in rat pituitary cells and that G_s_ (or a G_s_-like protein), G_13_ and Gβγ may participate in modulating these currents triggered by TRH (Miranda et al., 2005). There are some indications that rat TRH-R can modulate the function of NMDA ion channels (Kharkevich et al., 1991; Kinoshita et al., 1998). Interestingly, modulation of neuronal Na^+^ channels by TRH-R agonists has also been demonstrated in guinea pig septal neurons (López-Barneo et al., 1990). Besides modulating the function of nonselective cation channels, rat TRH-R appears to be able to affect electrogenic Na^+^/Ca^2+^ exchange (Parmentier et al., 2009). Interestingly, a recent study using mouse anterior pituitary cells has shown that transient receptor potential channel C and Orai1 may play an important role in the Ca^2+^ response triggered by TRH-R (Núñez et al., 2019). The effects of TRH-R activation on ion channel function are of great physiological importance, as these channels play a crucial role in modulating cell excitability.

An interesting topic of current molecular pharmacology research is the phenomenon of biased agonism, which has been well demonstrated at GPCRs. Different agonists can activate different signaling pathways *via* a one type of receptor because they stabilize different receptor conformations after binding (Kenakin, 2011). Intriguingly, not only do different agonists trigger different signal transduction pathways, but even a single agonist can initiate different signaling pathways *via* GPCRs. Biased receptor functionality is explained by the fact that each receptor can form many different specific conformations (active states) to which the agonist can bind (Franco et al., 2018). Importantly, biased responses can be elicited by biased ligands, biased receptors, or system bias, all of which can lead to preferential signaling through G proteins or β-arrestins (Smith et al., 2018). Biased agonism has been studied at several GPCRs, including G_q/11_-coupled receptors (Sanchez-Fernandez et al., 2013; Cabana et al., 2015; Mancini et al., 2015; Janovick et al., 2017; Teixeira et al., 2017). However, TRH-R has not received much attention in this regard. Our recent study in GH1 rat pituitary cells indicated possible biased TRH-R signaling induced by TRH and its analog taltirelin (Drastichova et al., 2022). This finding was confirmed by different phosphorylation patterns induced by these two ligands, and β-arrestin2 was found to play a key role in determining phosphorylation events after rat TRH-R activation. Extensive changes observed in many phosphosignaling pathways involving signal transduction *via* small GTPases, MAPK, Ser/Thr- and Tyr-protein kinases, Wnt/β-catenin, and members of the Hippo pathway suggest a previously unrecognized, wide-ranging impact of THR-R-initiated signaling. Future studies should pay more attention to TRH-R-mediated biased responses, which may help to provide better insights into the specific effects of different TRH-R ligands.

## Thyrotropin-releasing hormone receptor ligands and their pharmacological importance

TRH is the simplest hypothalamic hormone, a tripeptide composed of derivatives of the amino acids glutamate, histidine and proline. In particular, TRH is known to regulate the hypothalamic-pituitary-thyroid (HPT) axis, which plays a critical role in development, growth, and cell metabolism (Joseph-Bravo et al., 2015). TRH is secreted in a rhythmic pattern and controls the release and production of thyroid-stimulating hormone (TSH), which controls iodine accumulation and thyroid hormone secretion (Nillni, 2010). Thyroid hormones affect a variety of biochemical reactions involved in the regulation of energy metabolism (Daimon et al., 2013). TRH stimulates the release of prolactin and growth hormone, among others (Galas et al., 2009; Kanasaki et al., 2015). Inappropriate changes in the secretion of these hormones may be associated with pathophysiological situations such as acromegaly, renal failure, liver disease, cancer, diabetes mellitus, anorexia and bulimia, schizophrenia or depression (Zarate et al., 1986; Harvey, 1990; Ohbu et al., 1995; Freeman et al., 2000). In addition to its involvement in controlling the HPT axis, TRH may also act as a neurotransmitter or neuromodulator in the CNS and periphery. The effects of TRH on the CNS are far-reaching and may offer tremendous therapeutic potential. TRH has been reported to be involved in the regulation of emotional states (Sun et al., 2009). Another study confirmed its possible role in modulating anxiety states (Gutierrez-Mariscal et al., 2008). TRH may play a role in memory formation (Molchan et al., 1990; Zarif et al., 2016; Watanave et al., 2018) and other behavioral processes (Arnold et al., 1991; Hara et al., 2009). There is growing evidence that TRH can be implicated in neurodegenerative diseases of aging, such as Alzheimer’s disease and Parkinson’s disease (Daimon et al., 2013; Mohammadi et al., 2021). In the past, this agent has been used to treat traumatic brain/spinal cord injuries (Hashimoto and Fukuda, 1990; Pitts et al., 1995) and certain CNS disorders, including epilepsy (Przewłocka et al., 1997; Tanaka et al., 1998) and amyotrophic lateral sclerosis (Caroscio et al., 1986). Because of its antidepressant and anti-suicidal properties, it is thought to prevent suicidal behavior (Marangell et al., 1997). However, the clinical use of TRH for the treatment of psychiatric or neurological disorders is currently rather limited. In addition to its multiple effects on the CNS, TRH may also have other physiological effects. Of particular interest is the inotropic effect of TRH, which has been described in rats with ischemic cardiomyopathy (Jin et al., 2004). However, the potential clinical utility of TRH is rather limited because of its short half-life, low lipophilicity (low CNS and intestinal permeability) and some undesirable endocrine side effects. Therefore, great efforts are being made to find new analogs of TRH with better pharmacokinetic and pharmacodynamic parameters (Khomane et al., 2011). Taltirelin seems to be very promising in this regard and could have a positive effect on the treatment of various neurological disorders, e.g., obstructive sleep apnea (Liu et al., 2020).

TRH mimetics have been shown to be useful in the treatment of spinocerebellar degeneration (Shimizu et al., 2020; Ijiro et al., 2022), amyotrophic lateral sclerosis (Hawley et al., 1987), spinal muscular atrophy (Kat o et al., 2009), prolonged disturbance of consciousness due to aneurysmal subarachnoid hemorrhage (Shibata et al., 2019), Parkinson’s disease (Zheng et al., 2018a; Zheng et al., 2018b), epilepsy (Rajput et al., 2009; Sah et al., 2011), psychiatric disorders with underlying inflammatory processes (Kamath, 2012), and possibly depression (Duval et al., 2021). The effects of TRH and its analogs on the CNS are usually achieved in cooperation with the modulatory effects of neurotransmitters such as glutamate, dopamine, norepinephrine, acetylcholine, serotonin, or GABA. The close association of TRH-R-mediated effects with dopaminergic and cholinergic signaling appears to be crucial in the potential use of TRH and its analogs for the treatment of neurodegenerative diseases. Some of the functions of TRH in the context of pathological aging and neurodegeneration, and the potential of TRH and TRH mimetics for the treatment of neurodegenerative diseases have been discussed elsewhere (Daimon et al., 2013; Kelly et al., 2015).

There is growing preclinical evidence that activation of TRH-R can protect cells from a variety of deleterious influences. There are some indications that TRH and its analogs may have certain neuroprotective effects (Faden et al., 2005a; Faden et al., 2005b; Veronesi et al., 2007; Jantas et al., 2009; Zheng et al., 2018b), but the exact mechanism of these beneficial effects is not yet fully understood. One possible explanation for the *in vivo* neuroprotective effects of TRH likely involves several factors, including inhibition of the effects of glutamate, endogenous opioids, platelet activating factor, or leukotrienes, among others, which have a role in neurodegenerative damage. TRH may be able to help restore cellular bioenergetics and increase blood flow to the brain (Faden et al., 2005a). The neuroprotective action of TRH and its analogs against damage induced by various excitotoxic, necrotic, or apoptotic agents in primary cortical neurons appears to be independent of caspase-3 and does not involve pro-survival (PI3K/Akt and MAPK/ERK1/2) and pro-apoptotic (GSK-3β and JNK) signaling pathways (Jantas et al., 2009). Additionally, the data from these experiments suggest a possible involvement of calpain inhibition in TRH-R-mediated neuroprotective effects in the glutamate model of neuronal cell death. Interestingly, TRH receptor-mediated signaling need not directly exert cytoprotective effects on cells affected by agonist stimulation. It was reported that the TRH analog cerulein acts in the CNS to protect against acute pancreatitis through vagal and nitric oxide-dependent pathways (Yoneda et al., 2005). Similarly, central TRH may apparently play an important role in central vagal regulation of gastric cytoprotective mechanisms (Taché and Yoneda, 1993) and hepatic cytoprotection (Yoneda et al., 2003).

Possible antiapoptotic effects of TRH have also been reported. They were observed in both cellular and animal models (Drastichova et al., 2010; Koo et al., 2011; Luo et al., 2013). The mechanism of neuroprotective and antiapoptotic properties of TRH and its analogs might involve induction of ERK1/2 (Kanasaki et al., 2000), the PI3K/Akt pathway and Bcl-2 (Jaworska-Feil et al., 2010) and/or inhibition of monoamine oxidase B (Zheng et al., 2018b). On the other hand, the role of TRH in inducing apoptosis in pancreatic beta cell precursors has also been described (Mulla et al., 2009). Thus, the effects of TRH are likely to depend on the particular cell type and experimental conditions or physiological context.

## Conclusion and future directions

TRH is known to exert both neuroendocrine and extrahypothalamic effects, which are of great importance for the normal functioning of all vertebrates. Intensive research in recent decades yielded numerous publications on the biochemistry of TRH receptor-mediated signaling, as well as on the physiology, pharmacology, and potential therapeutic effects of TRH and its analogs. Most of the pioneering studies on the molecular mechanisms of TRH-R signaling have been conducted in the 1990s and the first decade of the 21st century. Although the basic principles of TRH-R-mediated signaling are known, there are still many unresolved issues. The role of di- and/or oligomerization of the receptor and the formation of protein complexes of TRH-R with other signaling partners should be further investigated in future studies to describe more precisely the molecular mechanisms of TRH-R-mediated signal transduction. The principles governing TRH-R trafficking are not well understood, and it is unclear how phosphorylated and dephosphorylated receptors are sorted in endosomes. TRH-R was originally considered to be a prototypical “calcium-mobilizing” GPCR. However, the signaling pathways triggered by TRH-R have proven to be more complex than anticipated. In addition to its interaction with different classes of G proteins, TRH-R apparently can also transduce signals through its diverse β-arrestin interactions in ways not yet fully recognized. Activation of TRH-R can apparently initiate various intracellular signaling pathways and lead to different responses depending on the cell type and specific experimental conditions. Some recent data suggest that biased agonism should be taken into consideration when characterizing TRH-R function and post-receptor signaling. These findings may open new avenues for the development of novel TRH analogs capable of controlling specific aspects of receptor function and potentially useful for therapeutic purposes. Research into the fundamental principles of TRH-R-mediated signaling deserves continued attention because of the important role this signaling system plays in maintaining a normal state of health. In the long term, these efforts are likely to have significant translational value, as the medical use of TRH and related drugs appears rational and warranted.
